# Comparative Genomics Provides Insights Into the Marine Adaptation in Sponge-Derived *Kocuria* *flava* S43

**DOI:** 10.3389/fmicb.2018.01257

**Published:** 2018-06-08

**Authors:** Wei Sun, Changrong Liu, Fengli Zhang, Mingzhu Zhao, Zhiyong Li

**Affiliations:** ^1^State Key Laboratory of Microbial Metabolism, School of Life Sciences and Biotechnology, Shanghai Jiao Tong University, Shanghai, China; ^2^Instrumental Analysis Center, Shanghai Jiao Tong University, Shanghai, China

**Keywords:** marine sponge, *Kocuria flava*, environmental adaptation, comparative genomics, third generation sequencing (TGS)

## Abstract

Sponge-derived actinomycetes represent a significant component of marine actinomycetes. Members of the genus *Kocuria* are distributed in various habitats such as soil, rhizosphere, clinical specimens, marine sediments, and sponges, however, to date, little is known about the mechanism of their environmental adaptation. *Kocuria flava* S43 was isolated from a coastal sponge. Phylogenetic analysis revealed that it was closely related to the terrestrial airborne *K. flava* HO-9041. In this study, to gain insights into the marine adaptation in *K. flava* S43 we sequenced the draft genome for *K. flava* S43 by third generation sequencing (TGS) and compared it with those of *K. flava* HO-9041 and some other *Kocuria* relatives. Comparative genomics and phylogenetic analyses revealed that *K. flava* S43 might adapt to the marine environment mainly by increasing the number of the genes linked to potassium homeostasis, resistance to heavy metals and phosphate metabolism, and acquiring the genes associated with electron transport and the genes encoding ATP-binding cassette (ABC) transporter, aquaporin, and thiol/disulfide interchange protein. Notably, gene acquisition was probably a primary mechanism of environmental adaptation in *K. flava* S43. Furthermore, this study also indicated that the *Kocuria* isolates from various marine and hyperosmotic environments possessed common genetic basis for environmental adaptation.

## Introduction

Marine sponges are well known to be a rich source of diverse actinomycetes such as *Streptomyces*, *Salinispora*, *Micromonospora*, *Kocuria*, etc. (Taylor et al., 2007). Sponge-derived actinomycetes are distributed randomly in sponge hosts, and they are thought to be taken up from the flowing sea water by filtration and dwell in the mesohyl matrix of sponge hosts (Abdelmohsen et al., 2014). Accordingly, the actinomycetes are endowed with the ability to survive in both nutrient-poor sea water and nutrient-rich sponge mesohyl. They are physiologically or metabolically distinct from their terrestrial counterparts owing to their adaptation to marine environments. A number of isolates grow and differentiate faster on the medium prepared with sea water (Jiang et al., 2007). Some *Streptomyces* strains strictly require salt for their growth (Khan et al., 2011). A few isolates own several secondary metabolite biosynthesis gene clusters absent in their closely related terrestrial relatives. For instance, sponge-derived *Streptomyces* sp. GBA 94-10 and PVA 94-07 share 10 identical secondary metabolite gene clusters not present in their terrestrial relative *S. albus* J1074, most of which are responsible for encoding non-ribosomal peptide synthetases and polyketide synthases (Ian et al., 2014). To date, research interest has been largely focused on the diversity and natural products of marine sponge-derived actinomycetes, however, the mechanisms behind their environmental adaptation are still not well understood. With the rapid development of the second/third next generation sequencing techniques, genomics may provide new insights into the genetic basis for environmental adaptation in sponge-derived actinomycetes.

Over the past decades, great progresses have been made in understanding the genetic basis for marine adaptation in Gram-negative bacteria. The discovery of the sodium-pumping NADH dehydrogenase Nqr (Unemoto and Hayashi, 1993) and the associated genes *nqrA-F* (Mulkidjanian et al., 2008) provided the genetic link to sodium dependence in Gram-negative marine bacteria. Nqr can create an ionic motive force to generate ATP and drive other cellular processes (Schäfer et al., 2008). The gene acquisition of certain transporters represents another important marine adaptation mechanism. High abundance of ABC branched chain amino acid (BCAA) transporters was found in several marine *Roseobacter* strains (Moran et al., 2007), and the associated genes were detected in marine metagenomes as well (Morris et al., 2010). BCAAs can be converted into L-glutamate, which would help acidify the basic cytoplasm (Takami et al., 2002). Marine cyanobacterium *Synechococcus*, with greater capacity to transport Na^+^ than freshwater species, might adapt to the oligotrophic environment by using more sodium-dependent transporters than a model freshwater cyanobacterium (Palenik et al., 2003). In addition, intracellular accumulation of compatible solutes seems to be an alternative marine adaptation strategy. The marine adaption in *Novosphingobium* was suggested to be based on the organic osmolyte (ectoine) mechanism that was different from those reported in Gram-negative marine bacteria that exported Na^+^ via the sodium-pumping NADH dehydrogenase Nqr (Gan et al., 2013).

Initial attempts to provide the evidence of marine adaptation in actinomycetes concentrated in the obligate marine genus *Salinispora* (Penn and Jensen, 2012). Marine adaptation genes (MAGs) were identified from *S. tropica* and *S. arenicola* by comparative genomic analysis, and the loss of a mechanosensitive channel gene *mscL* was thought to result in the inability of *Salinispora* strains to grow in low osmotic environment. The finding was later confirmed by genetic complementation of *S. tropica* with *mscL* (Bucarey et al., 2012). By the genomic comparison of two sponge-derived *Streptomyces albus* isolates with their terrestrial relative *S. albus* J1074, several putative MAGs linked to electron transport and potassium uptake were identified (Ian et al., 2014). In addition, marine-derived *Streptomyces* subgroup was found to possess some common characteristics of marine adaptation on the basis of comparative genomics (Tian et al., 2016). In specific, marine streptomycetes possessed more functional genes and transporters than other streptomycetes to adapt to the cold, hyperosmosis, oligotrophy, and other marine environments (Tian et al., 2016). Nonetheless, among diverse actinomycete groups, only a few representative members belonging to *Salinispora*, *Streptomyces*, *Janibacter*, *Aeromicrobium*, *Rhodococcus*, and one unclassified species “marine actinobacterium PHSC20C1” have been studied on their marine adaptation to date (Penn and Jensen, 2012; Tian et al., 2016). To improve our knowledge of the mechanisms for environmental adaptation in marine actinomycetes, it is significant to investigate a wider range of actinomycete lineages.

Taxonomically, *Kocuria* belongs to the family Micrococcaceae and comprises a group of coccoid actinomycetes with high environmental adaptability. *Kocuria* species have been recovered from various habitats, particularly marine environments such as sediments (Bala et al., 2012; Jiang et al., 2015), seawater (Seo et al., 2009), sponges (Abdelmohsen et al., 2014), and corals (Mahmoud and Kalendar, 2016). In our efforts of isolating actinomycetes from sponges collected from South China Sea (Li and Liu, 2006; Sun et al., 2015), *Kocuria flava* S43 was isolated from a coastal sponge *Siphonochalina* sp. (Sun et al., 2015), which had 99.7% similarity in 16S rRNA gene sequence with *K. flava* HO-9041 from the air of Xinjiang, an inland province in China (Zhou et al., 2008). Evidently, the inland airborne *K. flava* HO-9041 represents a terrestrial microorganism whereas the sponge-derived *K. flava* S43 is a marine representative. These two sister organisms can serve as a model for gaining insights into marine adaptation in *K. flava*. The complete genome sequence of *K. flava* HO-9041 is currently available (Zhou et al., 2016). Furthermore, a few complete or draft genome sequences of marine *Kocuria* isolates are publicly available, which provided an opportunity to make comparison with *K. flava* S43 at the genomic level. In this study, *de novo* genome sequencing for *K. flava* S43 was performed by TGS, and the genetic basis for marine adaptation in *K. flava* S43 was revealed by comparative genomic analysis of several *Kocuria* isolates from various marine and terrestrial environments.

## Materials and Methods

### Strain and 16S rRNA Gene Phylogeny

*K. flava* S43 was previously isolated from the tissue of the marine sponge *Siphonochalina* sp., which was collected from Xincun Harbor (18.42°N, 109.97°E), the South China Sea. The 16S rRNA gene sequence of *K. flava* S43 was deposited to GenBank under the accession number: JX007971 (Sun et al., 2015).

The type strains of the genus *Kocuria* were retrieved from RDP database (Release 11.5) by using the hierarchy browser^1^. Furthermore, literature search for the novel *Kocuria* species validly published in recent years was performed to update the type strain pool. The 16S rRNA gene sequences of the currently described 26 *Kocuria* species and *K. flava* S43 were manually aligned using ClustalX 1.81 (Thompson et al., 1997). Phylogenetic analysis was conducted using the maximum likelihood method (Felsenstein, 1981) provided by the software package MEGA 6.06 (Tamura et al., 2013). The consistency of the tree was verified by bootstrapping (1,000 replicates) for parsimony (Felsenstein, 1985).

### Genomic DNA Extraction and Genome Sequencing

A single colony of *K. flava* S43 was inoculated to a 50-ml centrifuge tube with 10 ml of GYM4 artificial seawater (26.52 g NaCl, 5.228 g MgCl_2_6H_2_O, 3.305 g MgSO_4_, 1.141 g CaCl_2_, 0.725 g KCl, 0.202 g NaHCO_3_, 0.083 g NaBr, and 1 L distilled water) medium (10 g glucose, 4 g yeast extract, 4 g malt extract, and 1 L artificial seawater) (Schneemann et al., 2010). After incubation at 28°C and 180 rpm for 2 days, the culture was collected by centrifugation and its genomic DNA was extracted using a DNeasy^®^ Blood and Tissue Kit (Qiagen, United States). The integrity, concentration and purity of genomic DNA were detected by Agarose Gel Electrophoresis, Nanodrop and Qubit Fluorimeter. The genome was sequenced by the third generation sequencing (TGS), i.e., the Pacific Biosciences (PacBio) Single Molecule Real-Time (SMRT) sequencing technology. A whole-genome sequencing library was constructed using the SMRTcell Template Prep Kit (PacBio, United States) according to the manufacturer’s protocol, and the library was sequenced by using g-Tube on the PacBio RS II platform (PacBio).

### Genome Annotation and Comparative Genomic Analysis

*De novo* assembly of the sequences was performed using hierarchical genome assembly process (HGAP) version 3.0 (Chin et al., 2013). This whole-genome shotgun project was deposited at DDBJ/EMBL/GenBank under accession number LOMZ00000000. The version described in this paper was the first version, LOMZ01000000. The draft genome sequence of *K. flava* S43 was uploaded to NCBI and aligned with the deposited genomes using Basic Local Alignment Search Tool^2^.

The genome sequences of *K. flava* HO-9041 from inland air, *Kocuria* sp. SM24M-10 from the mucus of the coral *Mussismilia hispida* (Palermo et al., 2016), *K. indica* DSM 25126 from a marine sediment sample (Dastager et al., 2014), *K. rhizophila* P7-4 from the intestine of the fish *Siganus doliatus* (Kim et al., 2011) and *K. rhizophila* DC2201 from a soil sample (Takarada et al., 2008) were downloaded from NCBI^3^ and served as the reference genomes for subsequent analysis. Genome alignment of *K. flava* S43 and *K. flava* HO-9041 was carried out using MAUVE software (Darling et al., 2010). For rapid function-based comparison by the comparative tool in the SEED Viewer (Overbeek et al., 2014), all the six *Kocuria* genome sequences were annotated by using Rapid Annotation using Subsystem Technology (RAST) version 2.0^4^ (Aziz et al., 2008). Genome Browser was used to visualize gene arrangement and annotation.

The subsystem category distribution pattern was compared between the *K. flava* S43 and HO-9041 genomes. The subsystems with significant difference in feature counts were marked and further compared at a finer scale. The common genes with marked increase in number in the *K. flava* S43 genome were screened. Meanwhile, the function-based comparison was performed among the 6 *Kocuria* genomes, the genes common in at least three marine *Kocuria* isolates but absent in two terrestrial *Kocuria* representatives were also screened. All the screened genes were listed as putative MAGs and uploaded to NCBI for protein match using BLASTp. The original RAST annotations were manually checked according to the BLAST hits using the NCBI Prokaryotic Genomes Automatic Annotation Pipeline (PGAAP) (Angiuoli et al., 2008). The genes incorrectly annotated by RAST were excluded.

### Phylogenetic Analysis of Genes Potentially Relevant to Environmental Adaptation

The screened candidate genes were subjected to phylogenetic analysis to test for a shared evolutionary history with other bacteria or actinomycetes derived from marine and hyperosmotic environments. The top 15–20 BLASTp hits of each gene were downloaded from the NCBI protein database. Maximum likelihood phylogenies were constructed for each representative gene using the online tool MABL with default settings^5^ (Dereeper et al., 2008). The genes that were clustered in the clades totally or largely comprising the homologs from the isolates of marine or hyperosmotic sources were kept in the final MAG pool.

## Results

### Phylogeny and Genome Feature of *K. flava* S43

**FIGURE 1:**
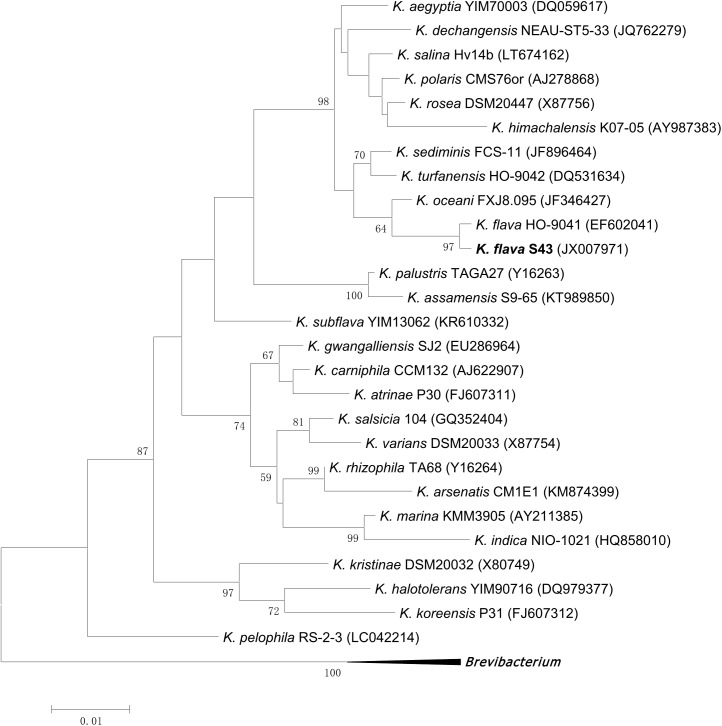
Maximum likelihood phylogenetic tree based on 16S rRNA gene sequences of *K. flava* S43 and all currently described *Kocuria* species. *Brevibacterium iodinum* DSM 20626 (X83813), *Brevibacterium linens* DSM 20425 (X7745), and *Brevibacterium casei* DSM 20657 (AJ251418) were used as the out-group. Only bootstrap values of being greater than 50% were shown on the tree.

Based on 16S rRNA gene sequence analysis, the phylogenetic position of *K. flava* S43 with respect to currently described *Kocuria* species was shown in **Figure 1**. *K. flava* S43 was clearly positioned within the genus *Kocuria* and formed a subgroup together with *K. flava* HO-9041.

**Table 1:** BLAST match of the *K. flava* S43 genome against the deposited bacterial genomes.

Contig no.	Contig size (bp)	Top BLAST hit	Protein-coding sequences	Coverage (%)	Identity (%)
Contig 1	3,548,480	*K. flava* HO-9041 genome	3,194	87	98
Contig 2	152,580	*K. flava* HO-9041 plasmid 1	136	23	91
Contig 3	140,250	*K. flava* HO-9041 plasmid 1	116	41	99
Contig 4	41,752	*K. turfanensis* HO-9042 plasmid 2	34	24	91
Contig 5	11,314	*K. flava* HO-9041 plasmid 1	10	49	95
Contig 6	2,899	No significant similarity	4		

A total of 99,440 reads of *K. flava* S43 were obtained, with a mean read length of 16,484 bp, providing 361.84× average reference coverage. The final assembly yielded six contigs, with the largest contig of 3,548,480 bp. The remaining five contigs were from 2,899 to 152,580 bp in length. BLAST matches indicated that the largest contig represented chromosome genome, four contigs were plasmid genomes and the smallest contig could not match any known genome (**Table 1**).

**FIGURE 2:**
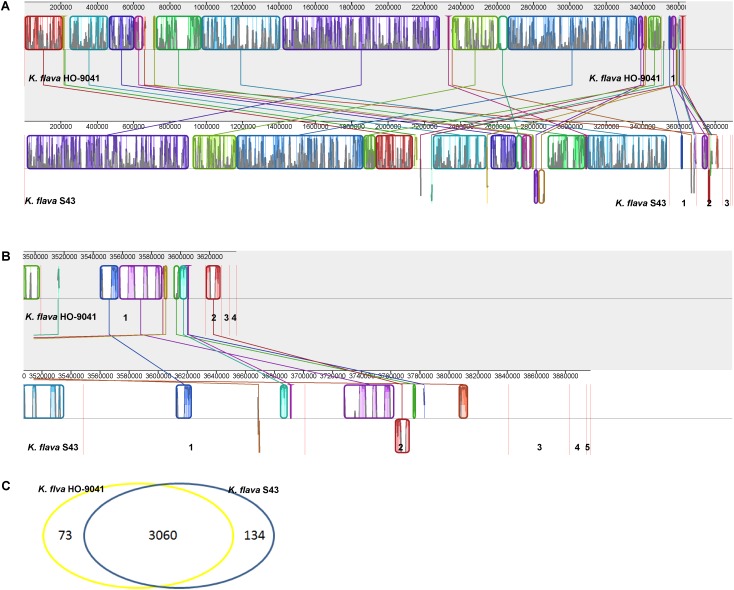
MAUVE alignment of the chromosome and plasmid genome sequences of *K. flava* S43 and *K. flava* HO-9041. The contigs were separated by red lines. When boxes had the same color, this indicated syntenic regions. Boxes below the horizontal line indicated inverted regions. Rearrangements were shown by colored lines. Scale was in nucleotides. **(A)** Chromosome with the boundaries marked by strain name. **(B)** Plasmid with the figures referring to different contigs. **(C)** Venn diagram showing the unique and common ORFs in 2 *K. flava* genomes.

The genome sequence of *K. flava* S43 was aligned with that of *K. flava* HO-9041. This alignment showed a high homology between *K. flava* S43 and *K. flava* HO-9041 (**Figure 2A**), supporting their close phylogenetic relationship as suggested by 16S rRNA gene phylogeny. Both genomes shared a large number of genomic traits, however, various translocations and inversions occurred in the genome of *K. flava* S43 relative to the reference genome. In addition, it appeared that gene transfer from plasmids to chromosomes happened in both genomes (**Figure 2B**). In specific, a few genes located in the *K. flava* S43 plasmid 1 and 2 showed a high homology with those in the *K. flava* HO-9041 genome and a few genes occurring in the *K. flava* HO-9041 plasmid 1 showed a high homology with those in the *K. flava* S43 genome, suggesting potential horizontal gene transfer.

Gene annotation of the draft genome sequence yielded 3,494 protein-coding sequences (CDSs), 49 tRNA and 11 rRNA (three 5S rRNA, four 23S rRNA, and four 16S rRNA) genes. In total, 3,194 CDSs, 48 tRNA, and 11 rRNA genes were located in the chromosome genome. The rest of the CDSs and one tRNA gene were distributed in the plasmid genomes.

### Genes Potentially Relevant to Marine Adaptation Based on Comparative Genomic Analysis

The comparison of the genomic characteristics between *K. flava* S43 and *K. flava* HO-9041 was presented in **Table 2**. The chromosome genome of *K. flava* S43 was slightly larger in size than that of *K. flava* HO-9041, suggesting the draft genome sequence was nearly complete. The same number of tRNA genes occurred in two genomes. Notably, although one copy was incomplete (5S rRNA missing and 23S rRNA partial) due to the draft sequence, the *K. flava* S43 genome owned one more copy of rRNA operon than the *K. flava* HO-9041 genome.

**Table 2:** Genomic characteristics of *K. flava* HO-9041 and *K. flava* S43.

Feature	*K. flava* HO-9041	*K. flava* S43
Genome size (bp)	3,504,335	3,548,480
GC content	74.2	74.2
Protein-coding genes (CDSs)	3,113	3,194
rRNA operons	3	4^∗^
tRNA genes	48	48

*^∗^One operon is incomplete*.

A Venn diagram showed the unique and common ORFs in two *K. flava* genomes (**Figure 2C**). The functional annotation of the unique ORFs in the *K. flava* S43 genome was shown in **Supplementary Table S1**. The comparison of the subsystem distribution between *K. flava* S43 and *K. flava* HO-9041 was demonstrated in **Table 3**. Significant difference was observed in the count of the genes categorized in six subsystems: respiration, potassium metabolism, virulence, disease and defense, phosphorus metabolism, cell wall and capsule, and nitrogen metabolism. The count of the genes related to respiration markedly increased in the *K. flava* S43 genome, which was due to the occurrence of the genes encoding respiratory complex I, terminal cytochrome d ubiquinol oxidase and the genes associated with the biogenesis of c-type cytochromes. Additional 4 *kdpD* (osmosensitive K^+^ channel histidine kinase) genes led to the higher number of genes related to potassium homeostasis in *K. flava* S43. Similarly, the counts of *czcD* (cobalt–zinc–cadmium resistance protein) and *copD* (copper resistance protein D) genes increased in *K. flava* S43, which were grouped into the virulence, disease, and defense subsystem. In the phosphorus metabolism subsystem, six *phoB* (phosphate regulon transcriptional regulatory protein) genes occurred in *K. flava* S43 whereas only three were present in *K. flava* HO-9041. In addition, the count of the genes categorized in cell wall/capsule and nitrogen metabolism markedly decreased in the *K. flava* S43 genome. In specific, more than 20 genes related to peptidoglycan biosynthesis were absent in *K. flava* S43. The number of the genes involved in nitrate and nitrite ammonification and encoding denitrifying reductase were less in *K. flava* S43 than in *K. flava* HO-9041.

**Table 3:** Subsystem distribution in the *K. flava* HO-9041 and *K. flava* S43 genomes.

Subsystem	*K. flava* HO-9041	*K. flava* S43
Cofactors, vitamins, prosthetic groups, pigments	221	221



Cell wall and capsule	67	40^∗^



Virulence, disease and defense	54	62^∗∗^



Potassium metabolism	16	21^∗∗^
Photosynthesis	0	0
Miscellaneous	53	58
Phages, prophages, transposable elements, plasmids	1	1
Membrane transport	77	79
Iron acquisition and metabolism	7	7
RNA metabolism	98	101
Nucleosides and nucleotides	98	104
Protein metabolism	224	229
Cell division and cell cycle	24	22
Motility and chemotaxis	3	3
Regulation and cell signaling	26	26
Secondary metabolism	10	10
DNA metabolism	66	67
Fatty acids, lipids, and isoprenoids	145	148



Nitrogen metabolism	38	31^∗^
Dormancy and sporulation	2	3



Respiration	49	78^∗∗^
Stress response	102	99
Metabolism of aromatic compounds	51	51
Amino acids and derivatives	320	321
Sulfur metabolism	33	32



Phosphorus metabolism	40	47^∗∗^
Carbohydrates	327	353

*^∗^The subsystems with significant decrease in feature counts of K. flava S43 were in light gray background rows*. *^∗∗^The subsystems with significant increase in feature counts of K. flava S43 were in gray background rows.*

**FIGURE 3:**
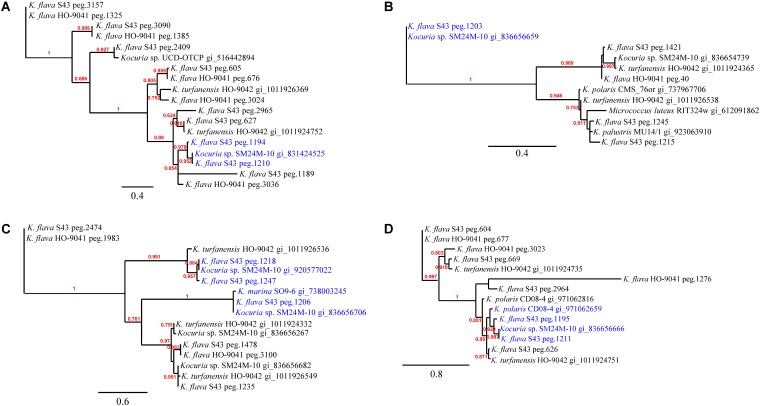
The phylogenies of the genes occurring in higher number in the *K. flava* S43 genome potentially relevant to marine adaptation. **(A)** *kdpD*, **(B)** *czcD*, **(C)** *copD*, **(D)** *phoB*. The clades totally comprising the homologs from the isolates of marine or hyperosmotic sources were colored blue. Midpoint rooting was used and likelihood values shown for each node. Only bootstrap values of being greater than 0.5 were shown on the tree. Scale bar represented changes per site.

Phylogenetic analyses of the *kdpD*, *czcD*, *copD*, and *phoB* genes in *K. flava* S43 were performed (**Figures 3A–D**). Apparently, the number increase of these genes was not due to gene duplication but gene gain of *K. flava* S43 or gene loss of *K. flava* HO-9041. Notably, each of the phylogenetic trees contained at least one clade totally comprising the homologs from the isolates of marine or hyperosmotic sources, such as *Kocuria* sp. SM24M-10 from a coral and *K. polaris* CD08-4 from a celiac disease patient (Chander et al., 2017), suggesting that a total of eight genes (two *kdpD*, one *czcD*, three *copD*, and two *phoB* genes) in such clades may be MAGs.

**FIGURE 4:**
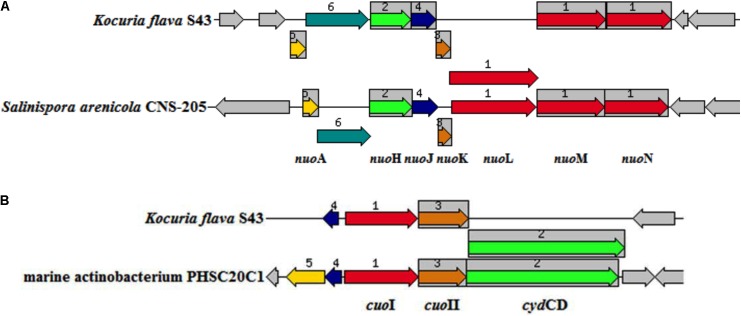
The arrangement of the operons potentially relevant to environmental adaptation in *K. flava* S43 and obligate marine actinomycetes. **(A)** nuo (NADH-ubiquinone oxidoreductase) operon, **(B)** cuo (cytochrome ubiquinol oxidase) operon. The chromosomal region of the focus gene (top) was compared with similar organisms. The graphic was centered on the focus gene, which was red and numbered 1. Sets of genes with similar sequence were grouped with the same number and color. Genes whose relative position was conserved were functionally coupled and shared gray background boxes.

A total of 13 genes (*nuoA*, *nuoH*, *nuoJ*, *nuoK*, *nuoL*, *nuoM*, *nuoN*, *cydA*, *cydB*, *cydCD*, *aqpZ*, and two *dsbA* genes) common in at least three marine isolates but absent in two terrestrial representatives were screened as putative MAGs. Seven *nuo* genes constituted a partial NDH-1 operon, which encoded a part of a proton-pumping NADH dehydrogenase. The *nuo* genes were previously identified as MAGs in *Salinispora* (Penn and Jensen, 2012). The organization of the operon in *K. flava S43* was consistent with that in *S. arenicola* CNS-205 (**Figure 4A**). Three *cyd* genes in a cluster were responsible for encoding cytochrome d ubiquinol oxidase subunit I, II and an ATP-binding cassette-type transporter, respectively. As one type of terminal respiratory oxidases, cytochrome d ubiquinol oxidase acted as the terminal acceptor of electron transport chains. The arrangement of the genes in the cluster was similar to that in the marine actinobacterium PHSC20C1 genome (**Figure 4B**). The *aqpZ* gene was located next to *cydCD*, which encoded an aquaporin, a specific water channel that facilitated the rapid influx/efflux of water, and the *dsbA* gene was responsible for encoding a periplasmic thiol/disulfide interchange protein, which was involved in the biogenesis of cytochrome C.

**FIGURE 5:**
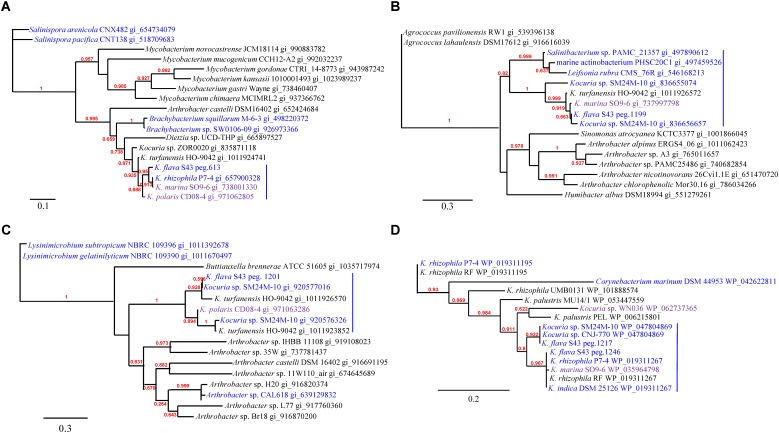
The phylogenies of the genes newly acquired in the *K. flava* S43 genome potentially relevant to environmental adaptation. **(A)** *nuoM*, **(B)** *cydB*, **(C)** *aqpZ*, **(D)** *dsbA*. Strains of marine, hyperosmotic, and terrestrial sources were colored blue, purple, and black, respectively. Midpoint rooting was used and likelihood values shown for each node. Only bootstrap values of being greater than 0.5 were shown on the tree. Scale bar represented changes per site.

The phylogenies of *nuoM* (**Figure 5A**), *cydB* (**Figure 5B**), *aqpZ* (**Figure 5C**), and *dsbA* (**Figure 5D**) indicated the presence of several clades totally or largely comprising the orthologs from the isolates of marine or hyperosmotic sources, suggesting that they shared evolutionary history with certain isolates derived from marine or other hyperosmotic environments.

The distribution and abundance of the MAGs in six *Kocuria* representatives were shown in **Table 4**. Besides four marine isolates, two strains from hyperosmotic environments, i.e., *K. polaris* CD08-4 from a celiac disease patient and *K. marina* SO9-6 from a copper iron sulfide mineral (Castro et al., 2015), were also included. Apparently, the MAGs were distributed in all or the majority of the analyzed *Kocuria* isolates though their abundance sometimes varied as the species, indicating that the *Kocuria* isolates possessed common genetic basis for marine adaptation. The result based on RAST annotation and comparison was consistent with the phylogeny test based on NCBI BLASTp.

## Discussion

Actinomycetes are widely distributed in marine and terrestrial environments. Besides a few obligate marine lineages such as *Salinispora* and *Marinispora*, the majority of the actinomycetes can survive in both marine and terrestrial habitats. Undoubtedly, marine actinomycetes acquire marine adaptive traits relative to their terrestrial counterparts. The genes relevant to marine adaptation can be identified by comparing the genomes of closely related marine and terrestrial microorganisms. Previous comparative genomic analyses of marine actinomycetes were performed mainly at the species or genus level (Penn and Jensen, 2012; Tian et al., 2016). In this study, comparative genomic analysis of *Kocuria* spp. was carried out at both strain and species level. Several MAGs were finally identified, indicating that comparative genomic analysis at the strain level was efficient and informative.

**Table 4:** The abundances of the MAGs in the investigated *Kocuria* spp. genomes.

Strain Source gene	*K. flava* S43 (HO-9041) sponge tissue	*Kocuria* sp. SM24M-10 coral mucus	*K. rhizophila* P7-4(DC2201) fish intestine	*K. indica* DSM 25126 marine sediment	*K. polaris* CD08-4 patient	*K. marina* SO9-6 copper miner
*kdpD*	9 (5)	6	2 (1)	2	6	1
*czcD*	4 (1)	3	2 (1)	3	1	3
*copD*	6 (2)	5	2 (1)	2	1	3
*phoB*	6 (3)	6	3 (1)	6	5	6
*nuo*	1 (0)	0	1 (0)	1	1	1
*cyd*	1 (0)	2	1 (0)	1	0	1
*aqpZ*	1 (0)	2	1 (0)	2	1	2
*dsbA*	2 (0)	1	2 (0)	1	0	1

*K. flava S43, Kocuria sp. SM24M-10, K. rhizophila P7-4 and K. indica DSM 25126 were representatives of various marine sources. K. polaris CD08-4 and K. marina SO9-6 were isolates from hyperosmotic environments. The numbers in brackets represented the gene number in its closely related terrestrial relative. kdpD, osmosensitive K^+^ channel histidine kinase; czcD, cobalt–zinc–cadmium resistance protein; copD, copper resistance protein; phoB, phosphate regulon transcriptional regulatory protein; nuo, NADH-ubiquinone oxidoreductase; cyd, cytochrome d ubiquinol oxidase; aqpZ, aquaporin; dsbA, periplasmic thiol/disulfide interchange protein*.

Gene gain is a major force driving bacterial evolution. The presence of the MAGs may be attributed to gene gain in marine *Kocuria* or gene loss in terrestrial *Kocuria*. In this study, the quantification of gene gain and loss was not performed. However, the gene arrangement in the *K. flava* S43 genome can provide a clue on gene gain or loss. The identified 21 MAGs are not scattered in the genome but concentrate in two genomic regions. One region consists of the seven *nuo* genes, and the other centers on the *cyd* genes. Particularly, a total of 12 genes (*kdpD*, *phoB*, *cycA*, *cycB*, *cycCD*, *aqpZ*, *czcD*, *copD*, *kdpD*, *phoB*, *dsbA*, *copD*) arrange in a cluster. On the upstream and downstream of the cluster, respectively, locates one mobile element, suggesting the gene cluster largely comprising the MAGs is acquired by horizontal gene transfer. Thus, gene acquisition potentially represents a primary mechanism of marine adaptation in *K. flava* S43.

The MAGs identified in the *K. flava* S43 genome potentially function in various ways. The respiratory complex I encoded by the nuo operon can create a proton-motive force for the generation of ATP and help maintain a proton gradient in seawater (Tokuda and Unemoto, 1982). The terminal respiratory oxidase encoded by *cydA* and *cydB* can act as the terminal acceptor of an electron transport chain and is therefore the key enzyme of respiration, and the ABC transporter encoded by *cydCD* can export cysteine, which is crucial for redox homeostasis in the periplasm (Pittman et al., 2002). The thiol/disulfide interchange protein encoded by *dsbA* can oxidize cysteine thiols of apocytochromes c and play a role in the biogenesis of c-type cytochromes (Peek and Taylor, 1992). Considering the high osmolarity in marine environments, the aquaporin AqpZ can provide the osmoregulation by mediating influx/efflux of water (Calamita, 2000), and the sensor kinase KdpD can osmotically regulate the potassium uptake (Jung et al., 1997). Surface seawater is known to contain trace amounts of heavy metals such as cadmium, copper, and zinc, the intracellular accumulation of which is harmful for the microorganisms. The copper resistance protein CopD can maintain the balance of copper by exporting copper and the cobalt–zinc–cadmium resistance protein CzcD can detoxify the periplasm by the export of toxic metal cations (cobalt, zinc, and cadmium) (Nies and Silver, 1995). Given the two-component response regulator PhoB can regulate phosphate assimilation in a sophisticated manner (Marzan and Shimizu, 2011), it probably facilitates the survival of *K. flava* S43 under phosphate-limiting niche. Taken together, the MAGs can help the marine *Kocuria* spp. adapt to oligotrophy and hyperosmolarity and resist to heavy metal ions.

The present study reveals that the *Kocuria* isolates from various marine and hyperosmotic environments have some common genomic features related to environmental adaptation. *K. flava* S43, *Kocuria* sp. SM24M-10, *K. indica* DSM 25126, *K. rhizophila* P7-4, *K. polaris* CD08-4, and *K. marina* SO9-6 were isolated from distinct habitats. The presence of some common MAGs suggests that the *Kocuria* isolates may adapt to respective niche in the same ways. The abundance of the MAGs in the *Kocuria* spp. varies as species, which can be attributed to the difference in geographic and environmental factors. It warrants attention that the *nuo* genes are missing in *Kocuria* sp. SM24M-10, and both *cyd* and *dsbA* are absent in *K. polaris* CD08-4. This phenomenon can be explained by their close associations with the hosts (coral mucus and patient), which provide nutrient-rich niches for the *Kocuria* isolates so they may reduce the energy requirement. Furthermore, *Kocuria* sp. SM24M-10, *K. indica* DSM 25126 and *K. marina* SO9-6 have more *aqpZ* genes than other *Kocuria* spp., implying the requirement of more aquaporins to regulate osmolarity. Taken together, these results indicate that the MAGs are not essential for the survival in marine environments. However, the presence of the genes potentially improves the environmental adaptability of the *Kocuria* isolates.

It was proposed that no common genetic basis for marine adaptation existed among different actinobacterial genera (Penn and Jensen, 2012). Our study shows that the MAG pool in marine *Kocuria* isolates markedly differs from those in *Salinispora* or marine *Streptomyces*, further supporting this suggestion. Specifically, over half the MAGs in *Kocuria* are grouped into electron transport systems, however, in *Salinispora* channels and pores account for more than half of the MAGs (Penn and Jensen, 2012). K^+^ transporters (Trk) and betaine/carnitine/choline transporters are highly rich in marine *Streptomyces* group (Tian et al., 2016). It is noteworthy that although the MAG pool varies significantly in gene content and abundance among different actinomycete genera, the type and function of the MAGs are almost consistent, i.e., respiration/electron transport system, cation and ABC transporters and channels/pores. The results suggest that the functional adaptation of diverse actinomycetes to marine environments is convergent. Previous analyses revealed that the mechanisms of marine adaptation in *Salinispora* spp. were fundamentally different from those reported for Gram-negative bacteria (Penn and Jensen, 2012). Our finding that the gene content in the MAG pool of *Kocuria* significantly differs from that of Gram-negative bacteria also supports this conclusion. The role of Nqr in Gram-negative marine bacteria seems to be replaced by that of a proton-pumping NADH dehydrogenase in marine actinomycetes. In contrast to the well-studied Gram-negative bacteria, much fewer researches have focused on Gram-positive bacteria. With increasing Gram-positive bacteria investigated from a genomic view, a clear picture of marine adaptation in Gram-positive bacteria will be obtained.

## Conclusion

This study reveals that *K. flava* S43 might adapt to the marine environment by increasing the number of the genes related to potassium homeostasis, phosphate metabolism and resistance to heavy metals, and acquiring the genes associated with electron transport and the genes encoding ABC transporter, aquaporin, and thiol/disulfide interchange protein. Gene acquisition is potentially a primary mechanism of environmental adaptation in *K. flava* S43. Furthermore, this study indicates that the *Kocuria* isolates from various marine and hyperosmotic environments have common genomic characteristics for environmental adaptation. The findings expand our knowledge of the genetic basis for marine adaptation in *Kocuria*.

## Author Contributions

ZL and WS conceived and designed the study. MZ was responsible for sequencing and assembly. WS, CL, and FZ performed data analyses. WS and ZL drafted the manuscript. All authors approved the final manuscript.

## Conflict of Interest Statement

The authors declare that the research was conducted in the absence of any commercial or financial relationships that could be construed as a potential conflict of interest.

## Footnotes

1 http://rdp.cme.msu.edu/

2 https://blast.ncbi.nlm.nih.gov/Blast.cgi

3 ftp://ftp.ncbi.nih.gov/genomes/genbank/bacteria/Kocuria

4 http://rast.nmpdr.org/

5 http://phylogeny.lirmm.fr/phylo_cgi/simple_phylogeny.cgi

## Supplementary Material

TABLE S1 The functional annotation of the unique ORFs in the *K. flava* S43 genome.
